# Predictors of persistent cytologic abnormalities after treatment of cervical intraepithelial neoplasia in Soweto, South Africa: a cohort study in a HIV high prevalence population

**DOI:** 10.1186/1471-2407-8-211

**Published:** 2008-07-25

**Authors:** Yasmin Adam, Cyril J van Gelderen, Guy de Bruyn, James A McIntyre, Diane A Turton, Neil A Martinson

**Affiliations:** 1 Department of Obstetrics and Gynaecology, Chris Hani Baragwanath Hospital, Johannesburg, South Africa; 2 University of the Witwatersrand, Johannesburg, South Africa; 3 Perinatal HIV Research Unit, University of the Witwatersrand, Johannesburg South Africa; 4 National Health Laboratory Service, Johannesburg, South Africa; 5 Johns Hopkins University School of Medicine, Baltimore, USA

## Abstract

**Background:**

In the presence of both HIV infection and cervical intraepithelial neoplasia (CIN), the risk of cancer development despite treatment may be greater. We investigated clinical predictors of persistent cytological abnormalities in women who had had a large loop excision of the transformation zone (LLETZ).

**Methods:**

Women with high grade squamous intraepithelial lesions or worse (HSIL), less severe abnormalities which persisted and any abnormality in women who are HIV-infected, were referred to the colposcopy clinic. HIV infection was ascertained by self-report. A LLETZ was performed on all patients with HSIL or higher on Papanicolaou (Pap) smear or colposcopy, LSIL or higher in patients who are HIV-infected, where the colposcopy is inadequate, and when there was a discrepancy between colposcopy and cytology by one or more grades. Women with abnormal follow-up smears were compared to those with normal smears. We examined the association between abnormal follow-up smears and demographic and clinical predictors using logistic regression

**Results:**

The median time between LLETZ and first follow-up Pap smear was rather short at 122 days. Persistent cytological abnormalities occurred in 49% of our patients after LLETZ. Predictors of persistence included the presence of disease at both margins and HIV infection. Among the latter, disease at the excision margins and CD4+ cell count were important predictors. In these women, disease at the endocervical margin, both margins, and disease only at the ectocervical margin were associated with increased odds of persistent abnormalities on follow-up cervical smear.

**Conclusion:**

We showed extremely high risk of cytological abnormality at follow-up after treatment more so in patients with incomplete excision and in the presence of immunocompromise. It remains uncertain whether recurrent CIN is a surrogate marker for invasive cervical cancer.

## Background

Cervical cancer is the second leading cancer in women after breast malignancy in South Africa (SA)[1]. The life-time risk of developing invasive cervical cancer (ICC) is 1 in 31 for South African women. The crude rates for ICC were 26.1 per 100 000 in 1999, with a corresponding age-standardized incidence rate of 28.7 per 100 000 and the risk increased with age, peaking at 136.4 per 100 000 in women between the ages of 65 and 69[2]. Worldwide, the ratio of mortality to incidence is 55% with a worse prognosis in developing countries[1].

Several studies from Sub-Saharan Africa have shown associations between HIV infection and cervical cancer [3-5]. Furthermore, in HIV-infected women, premalignant disease of the cervix is more frequent, of a higher grade, and progresses more rapidly[6]. The prevalence of abnormal Pap smears was between 50% and 75% in HIV-positive women in two studies in Sub-Saharan Africa[7,8] and in the Gauteng province of SA, where our clinic is situated cervical cytological abnormalities are found in 13.7% of all women. This is a surprisingly high prevalence even allowing for a high HIV infection rate (30.8% in antenatal attendees in the same community[9]).

The prevention of ICC includes screening for pre-malignant disease, treatment and follow-up of treated patients. A National Screening Policy, whereby state health will pay for three cervical smears in a life-time commencing at the age of thirty, was instituted in SA in 2001[10]. By the year 2006, 5.2% of screened women were being referred for colposcopy in the Johannesburg Metropolitan area compared to 3.5% in the UK[11,12].

Local excision and ablation of the cervix is the standard treatment for CIN2-CIN3. Although treatment reduces the risk of subsequent ICC[13,14], these women remain at a 5-fold increased risk of ICC as compared to the general population[15]. Follow-up for recurrent CIN is therefore an important aspect of the prevention of cancer program. The identification of meaningful risk factors for persistence will not only pinpoint, those who need to be followed up more actively after treatment of a premalignant lesion, but may designate those who may safely be followed at less sophisticated facilities, and perhaps at less frequent intervals. The objective of this study was to document rates and predictors of persistence of premalignant cervical lesions in women who had had a large loop excision of the transformation zone (LLETZ) at a "see and treat" colposcopy clinic in Soweto, South Africa.

## Methods

We followed up a cohort of women attending the colposcopy clinic at Chris Hani Baragwanath Hospital in Soweto, South Africa. The clinic is a referral site for women with abnormal cervical smear results who predominantly live in Soweto but also are referred from the southern parts of the Province. Cytological cervical smears are reported according to the 2001 Bethesda System Terminology[16]. These reports include: invasion, high or low grade squamous intraepithelial lesion (HSIL or LSIL), atypical glandular cells of undetermined significance (AGUS), atypical squamous cells suggesting HG (ASC-H) or atypical cells of undetermined significance (ASCUS).

Initially HIV status was ascertained by self-report. Voluntary counseling and testing (VCT) was only started in November 2006. HIV positive results were confirmed by clinical notes of women who self-reported being HIV positive. The status of those patients who said that they were HIV negative was recorded as such if the test was done within the last 6 months. However the result was not always confirmed. Those who said they tested negative more than 6 months previously was recorded as unknown.

We offer a "see and treat service" where such patients are offered immediate diagnosis and treatment with colposcopy and LLETZ [17]. A LLETZ is performed on all patients with HSIL or higher on cervical smear or CIN 2 or higher on colposcopy, where the colposcopy is inadequate, or when there is a discrepancy between colposcopy and cytology by one or more grade. HIV-infected women with L SIL or more on cervical smear and CIN 1 or higher on colposcopy are treated due to the higher progression[6] and recurrence in these women.

Patients were followed up at six-monthly intervals. Those women who had not returned by 6 months were contacted by mail or telephone; if there was no response, a letter was hand delivered by a research worker to the listed home address to encourage follow up or to ascertain survival. The total number of patients that were lost to follow-up was 420(41.3%). The loss to follow-up according to HIV status was 176(42%) for HIV positive women, 109(26%) for HIV negative women (self-reported) and 135(32%) for the women who did not know their status.

Owing to the study design, we cannot differentiate between persistence and recurrence of abnormal lesions. For the purpose of this study, therefore, any abnormal cytology after LLETZ was defined as being persistent. Ethical approval for this study was obtained from the Human Research Ethics Committee (medical) of the University of the Witwatersrand.

### Statistical analyses

Women with abnormal follow-up smears were compared to those with normal smears using t-tests for continuous variables and Chi-square tests for categorical variables. We examined the association between abnormal follow-up smears and demographic and clinical predictors using logistic regression. Bivariate association between the outcome of interest and predictor variables was first performed. Multivariate association was then examined in a stepwise logistic regression including only those variables with a bivariate *p*-value of 0.2 or greater. Two separate models were investigated. The first included all participants, and included HIV status as a predictor variable. The second model restricted the analysis population to women who were HIV-infected.

## Results

**Figure 1:**
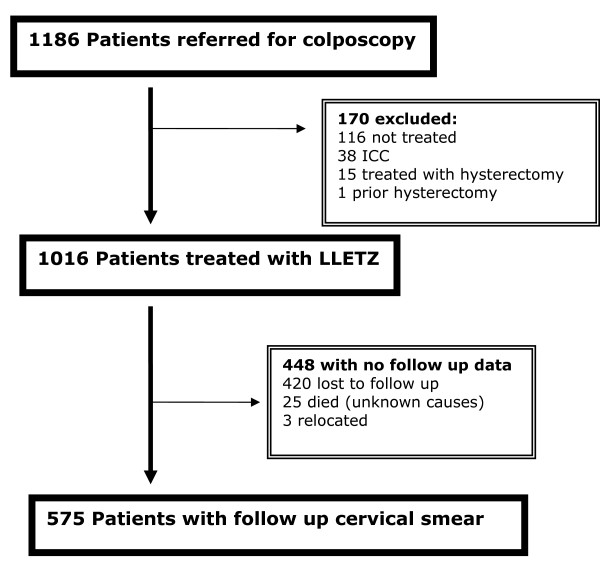
Study schema.

Between April 2003 and November 2006, 1186 women were referred to the colposcopy clinic at Chris Hani Baragwanath Hospital (CHB) in Soweto. Of these, 1016 had a LLETZ performed and 575 (57%) women returned to the clinic for a follow-up cervical smear and were included in this analysis (Figure 1). LLETZ treatment was not offered to 170 women: 116 of whom were not eligible for treatment at the time (pregnancy, normal cervix on colposcopy, CIN 1 in immunocompetent women), 38 had invasive cervical cancer, and 15 required hysterectomy for other reasons. 290 women had normal follow-up cervical smears(50.4%) and 285(49.4%) women had persistently abnormal smears after LLETZ. We included any abnormal Pap smear after treatment in the group classified as persistence, that is any grade of abnormality and any Pap smear after treatment.

The median time from the date of LLETZ to second cervical smear was 122 days (interquartile range (IQR), 71 – 160 days). There was no difference in the average duration between LLETZ and follow-up smear for those with an abnormal(136.1 days) and those with a normal follow-up smear(136.3 days). There were 80 women who had a second procedure in the group with persistence (LLETZ, cone or hysterectomy). Second treatments were postponed in some women to allow initiation of ARV therapy or in women who were not medically fit for surgery. We did not perform colposcopic examinations on the women with normal follow-up cytology. The cytologic abnormalities noted on the follow up smears are listed in Table 1.

**Table 1:** Results Of Follow Up Cytologic Smears Following Large Loop Excision Of The Transformation Zone (LLETZ), Chris Hani Baragwanath Hospital, Soweto 2003 – 2006.

**Normal cervical cytology at follow-up**	**290 (50.4%)**
**Cytological abnormalities at follow up**	**285 (49.6%)**
ASCUS	18 (6.3%)
L SIL	136 (47,6%)
ASC-H	5 (1.8%)
AGUS	1 (0.4%)
H SIL	123 (43.2%)
Invasion	2 (0.7%)

Four women had Microinvasion on histology after LLETZ, one fell pregnant, 2 refused hysterectomy, and one had a hysterectomy 6 months after the LLETZ. There was no dysplasia, only features of HPV infection on the histology. All 4 cases were a stage 1a1.

Age, HIV infection, histology of the excised portion and the presence of disease at an excision margin were notable differences between those women with persistent cytologic abnormalities and those with normal follow up smears (Table 2). We examined associations of women having an abnormal cervical smear at their follow-up visit with various clinical predictors using logistic regression (Table 3). Self-reported HIV status and the presence of disease at both margins were strongly associated with persistent cytologic abnormalities after LLETZ. Restricting the analysis only to those women who self -reported themselves to be HIV-infected, increasing CD4 count (the CD4 count was the count within 6 months of the treatment date) was found to be associated with lower odds of abnormal follow-up smear results, but having either the endocervical or both margins involved was highly associated with persistent cytologic abnormalities. In addition, the presence of CIN3 on the excised tissue resulted in a marked increase in the risk of recurrence compared to CIN1 (OR = 3.8, CI = 1.1–13.1) (Table 4).

**Table 2:** Characteristics Of Women With Normal Or Abnormal Follow Up Cytologic Smears Following LLETZ, Chris Hani Baragwanath Hospital, 2003 – 2006.

	Abnormal* (n = 285)	Normal* (n = 290)	***p*-value**
Age, mean (sd)	35.1 (8.0)	37.5 (8.9)	0.001
Parity, mean (sd)	2.7 (1.02)	2.98 (0.97)	0.004
Contraceptive method, n (%) reporting use			
Norethisterone oenanthate	32 (11.2)	43 (14.8)	0.200
Depomedroxyprogesterone acetate	29 (10.2)	48 (16.6)	0.025
Intra-uterine contraceptive device	3 (1.1)	4 (1.4)	0.721
Progestogen-only pill	3 (1.1)	7 (2.4)	0.212
Combined oral contraceptive	20 (7.0)	26 (9.0)	0.389
Sterilization	9 (3.2)	19 (6.6)	0.059
HIV status, n (%) self-reporting status			<0.0001
Negative	37 (13.0)	112 (38.6)	
Positive	184 (64.6)	82 (28.3)	
Unknown	64 (22.5)	96 (33.1)	
CD4, n (%)			<0.0001
<200	91 (55.5)	23 (31.1)	
200 – 499	59 (36.0)	33 (44.6)	
>499	14 (8.5)	18 (24.3)	
On antiretroviral therapy	68 (23.86)	21 (7.24)	<0.0001
Baseline smear result, n (%)			0.947
H SIL	216 (75.8)	196 (67.6)	
L SIL	34 (11.9)	33 (11.4)	
ASCUS	12 (4.2)	12 (4.1)	
Histology of excised transformation zone, n (%)	270	255	0.139
Normal	6 (2.2)	7 (2.8)	
CIN I	18 (6.8)	28 (11.3)	
CIN II	116 (43.9)	90 (36.3)	
CIN III	129 (48.9)	126 (50.8)	
Microinvasion	1 (0.4)	4 (1.6)	
Presence of dysplasia at the margin of excised transformation zone	253	243	<0.0001
Both	35 (13.8)	14 (5.8)	
Ectocervical	84 (33.2)	46 (18.9)	
Endocervical	35 (13.8)	35 (14.4)	
Margins clear	99 (39.1)	148 (60.9)	

* Numbers in each category may not sum to the total for the column owing to missing values.

**Table 3:** Logistic regression using data of all women whose HIV status was known showing associations with persistence of an abnormal smears following LLETZ (n = 295).

	**Univariate ** **Odds Ratio ** **(95% CI)**	**Multivariate ** **Odds Ratio ** **(95%CI)**	***p*-value**
**HIV infection**	6.8 (4.3 – 10.7)	8.2 (4.2–15.8)	<0.0001
**Disease at excision margin of biopsy specimen**			
No disease at the excision margins	Referent	Referent	-
Disease at the Ectocervical margin	2.1 (1.4 – 3.2)	2.2 (1.2–4.1)	0.013
Disease at the Endocervical margin	0.96 (0.6 – 1.6)	2.1 (0.9–5.5)	0.09
Both endocervical and ectocervical margins	2.6 (1.4 – 5.0)	10.7 (3.0–37.4)	<0.0001
**Histology**			
CIN1	Referent	Referent	-
CIN2	2.0 (1.0 – 3.9)	2.0 (0.8–5.1)	0.17
CIN3	1.6 (0.8 – 3.0)	2.2 (0.8–6.0)	0.12
Micro-invasion	0.4 (0.04 – 3.8)	2.00 (0.04 – 96.7)	0.73
**Age > 35 years (median age)**	0.64 (0.5 – 0.9)	0.58 (0.33 – 1.0)	0.06

(n = 295).

**Table 4:** Logistic regression assessing associations with persistence of abnormal smears following LLETZ: restricted to HIV-infected women (n = 196).

	**Univariate ** **Odds Ratio ** (95% CI)	**Multivariate ** **Odds Ratio ** (95%CI)	***p*-value**
**CD4 category (cells/mm^3^)**			
<200	Referent	Referent	-
200–499	0.45 (0.24 – 0.85)	0.4 (0.2–0.8)	0.017
≥500	0.2 (0.085 – 0.45)	0.1 (0.01–0.2)	0.000
**Disease at excision margins**			
Margins not involved	Referent	Referent	-
Ectocervical margin positive	1.7 (0.93 – 3.3)	2.0 (0.9–4.6)	0.089
Endocervical margin positive	2.2 (0.8 – 5.8)	5.6 (1.3–24.8)	0.024
Both endo- and ectocervical margins involved	2.5 (0.86 – 7.1)	4.3 (0.9–19.2)	0.059
**Histology of excised transformation zone**			
CIN1	Referent	Referent	-
CIN2	2.1 (0.8 – 5.3)	2.5 (0.8–7.8)	0.116
CIN3	2.5 (0.96 – 6.4)	3.8 (1.1–13.1)	0.037

## Discussion

We report high rates of persistence of cytological abnormalities on a follow-up cervical smear after initial treatment using LLETZ particularly in women self reporting as being HIV-infected. Cytological abnormalities after LLETZ were eight times more frequent in women who self reported as being HIV infected. However, in HIV-infected women with a CD4 count of ≥ 500 cells/mm^3^, this risk was halved when compared to women with a CD4 count of <200 cells/mm^3^. Dysplasia at both the endocervical and ectocervical excision margins markedly increased the risk of persistence as compared to absent dysplasia at the margins (OR = 10.7, CI = 3.0–37.4). In univariate analysis, the risk of persistence with disease present at either excised margin was double that of no disease at the margin.

The association of HPV and cervical cancer has been established[18]. Impaired cell mediated immunity is a risk factor for HPV infection and CIN[19]. Observational studies have shown an association between CIN and co-infection with HPV and HIV [20]. In this study it was not possible to distinguish between the effect of HIV infection and changes in immunity as would be expected in women on ARV therapy.

Of the 266 women who were HIV-infected, 89(33%) of them were on ARV therapy and the number of women taking ARV's were significantly higher in the patients with subsequent persistence. However, duration of and response to treatment would be required to assess ARV therapy as an independent variable predicting persistence and we did not collect this information. Longitudinal studies of detection of oncogenic HPV types and cytologic dysplasia among HIV infected women on HAART indicate that ARV therapy may result in clearance of HPV and regression of low grade lesions [21-23].

The histological report contributed to the identification of women at higher risk of persistence. Firstly, univariate analysis of the entire group suggests that if the excised biopsy specimen had margins involved, risk of persistence was significantly higher. In multivariate analysis, this effect was marked. However, because of the relatively small numbers of women in the multivariate analysis, the ability to draw conclusions from this finding is somewhat limited. Secondly, histological reports of CIN-2 and CIN-3 increased the risk of persistence compared to CIN-1. In addition, a surprising finding in this study was that increasing age appeared to be protective against persistence as the reported risk of cervical malignancy increases with age, peaking at 136.4 per 100 000 in women between the ages of 65 and 69 in South Africa[2].

In this study, we did not see an association between persistent cytologic abnormalities and use of hormonal contraception. This was not thoroughly investigated. Resumption of safe sex practices is associated with regression in women with established neoplasia[24,25]. Condom usage has previously been shown to be higher in women who are HIV positive[26]. This could not be explored in this study, again limited by the ascertainment of condom use.

Potential sources of bias in this study include its operational nature. In this setting, not all eligible women were responsive to visit reminders or other measures to encourage follow up.

In most of the women in this report, we relied on self-report for HIV serostatus. Even though HIV positivity could be confirmed by clinical notes, women who reported an HIV negative status could not always be confirmed. In South Africa the stigma of being HIV infected causes many people to deny their HIV infection. It is probable, therefore that misclassification would err in the direction of women reporting themselves as HIV-negative, and, if this is so, the associations we have drawn may be an underestimate. Compliance rates for treatment of SIL range widely, 30%–73% [27,28], depending on the setting (e.g., in a developing vs. an inner city population). Furthermore, we did not record the size of the lesion on colposcopy nor size of the excised lesion. We did not record the smoking history or a detailed sexual history and therefore were unable to adjust for them in the regression models. The numbers of abnormal cervical smears may have been inflated by including those that do not traditionally require immediate treatment. However, an audit at our clinic in 2006 showed that patients with cervical smears demonstrating L SIL had CIN2/3 on histological examination of excised biopsy specimens in 68.5%[29]. HPV testing is not routinely available in our clinical setting.

Factors previously shown to predict persistence of premalignant cervical lesions include the presence of disease at the margins[30], the grade of CIN [30], oncogenic HPV types[31], HPV variants[30], immunocompetence unrelated to HIV factors, age, smoking[32], and sexual behaviour[33]. In some studies, clear margins did not guarantee eradication [34-36]. Lesion size has a variable association with persistence[37] and the presence of disease at the excision margins may be related to lesion size or excision technique but we did not see any difference in rates of persistence between the two operators (data not shown). In addition, the choice of treatment modality impacts on rates of persistence. Among HIV-infected women, persistence of SIL after Cryotherapy has been reported to be between 48% and 100%, and after conization between 18% and 71%[38,39].

More intensive, long-term follow-up of HIV infected women after LLETZ is warranted and provider-initiated HIV testing should therefore be offered in any patient with an abnormal cervical smear result, to enable appropriate follow up arrangements. Furthermore, our data suggests that complete excision of the transformation zone should be the objective of surgical interventions, despite the potential for an increase in associated procedural complications. In our setting, it appears reasonable for HIV negative women who have clear excision margins to have less intensive follow up, at the level of a community health centre.

## Conclusion

We have observed extremely high rates of persistence of CIN in HIV-infected women and in women with disease at the excision margins following LLETZ. More intensive follow up of women with margins involved by CIN and especially women who are HIV infected with CD4 count under 500 is warranted. However, it remains unclear whether persistent CIN identifies those women at risk for progression to ICC.

## Competing interests

The authors declare that they have no competing interests.

## Authors' contributions

YA conceived of the study, performed the surgical treatments, and participated in the design of the study and drafting of the manuscript. CJvG performed the surgical treatments and contributed to the drafting of the manuscript. GdB participated in the drafting of the manuscript and performed the statistical analysis. DAT performed and supervised the histological analysis of all the specimens. JAMcI contributed to the drafting of the manuscript. NAM contributed to the design and drafting of the manuscript. All authors read and approved the final manuscript.

## Pre-publication history

The pre-publication history for this paper can be accessed here:



## References

[B1] Parkin DM, Bray F, Ferlay J, Pisani P (2005). Global Cancer Statistics, 2002. CA: a cancer journal for clinicians.

[B2] Mqoqi N, Kellett P, Sitas F, Jula M (2004). Incidence of histologically diagnosed cancer in South Africa, 1998-1999. National cancer registry.

[B3] Newton R, Ziegler J, Beral V, Mbidde E, Carpenter L, Wabinga H, Mbulaiteye S, Appleby P, Reeves G, Jaffe H (2001). A case-control study of human immunodeficiency virus infection and cancer in adults and children residing in Kampala, Uganda. Int J Cancer.

[B4] Gichangi PB, Bwayo J, Estambale B, De Vuyst H, Ojwang S, Rogo K, Abwao H, Temmerman M (2003). Impact of HIV infection on invasive cervical cancer in Kenyan women. AIDS.

[B5] Sitas F, Pacella-Norman R, Carrara H, Patel M, Ruff P, Sur R, Jentsch U, Hale M, Rowji P, David S, Connor M, Bull D, Newton R, Valerie B (2000). Epidemiology and Cancer Prevention. The spectrum of HIV-1 related cancers in South Africa. Int J Cancer.

[B6] Wright TCJ, Ellerbrock TV, Chiasson MA, Van Devanter N, Sun XW (1994). Cervical intraepithelial neoplasia in women infected with human immunodeficiency virus: prevalence, risk factors, and validity of Papanicolaou smears. New York Cervical Disease Study. Obstet Gynecol.

[B7] Moodley J, Hoffman M, Carrara H, Allan B, Cooper D, Rosenberg L, Denny LE, Shapiro S, Williamson A (2006). HIV and pre-neoplastic lesions and neoplastic lesions of the cervix in South Africa: a case control study. BMC Cancer.

[B8] Parham G, Sahasrabuddhe V, Mwanahamuntu M, Shepherd B, Hicks M, Stringer E (2006). Prevalence and predictors of squamous intraepithelial lesions of the cervix in HIV-infected women in Lusaka, Zambia. Gynecol Oncol.

[B9] DoH SA National HIV and syphilis antenatal sero-prevalence survey in South Africa, 2006.

[B10] National guidelines for cervical cancer screening in South Africa,2000. http://www.doh.gov.za/docs/facts-f.html.

[B11] DoH  (2006). Cervical Cancer Screening: PHC Facilities. Precancerous and Cancerous Cervical smears, July-October 2006.

[B12] Cervical Screening Programme England: 2005-2006.

[B13] Laara A, Day NE, Hakama M (1987). Trends in mortality from cervical cancer in the Nordic countries: association with organized screening programs. Lancet.

[B14] Boyes DA, Worth AJ, Anderson GH (1981). Experience with cervical screening in British Columbia. Gynecol Oncol.

[B15] Soutter WP, Sasieni P, Panoskaltsis T (2006). Long-term risk of invasive cervical cancer after treatment of squamous cervical intraepithelial neoplasia.. Int J Cancer.

[B16] Solomon D, Davey D, Kurman R, Moriarty A, O'Connor D, Prey M, Raab S, Sherman M, Wilbur D, Wright TJ, Young N (2002). The 2001 Bethesda System.Terminology for reporting results of cervical cytology.. JAMA.

[B17] Cárdenas-Turanzas M, Follen M, Benedet JL, Cantor SB (2005). See-and-treat strategy for diagnosis and management of cervical squamous intraepithelial lesions. Lancet Oncol.

[B18] Bosch FX, Lorincz A, Munoz N, Meijer CJ, Shah KV (2002). The causal relation between human papillomavirus and cervical cancer. J Clin Pathol.

[B19] Sun XW, Kuhn L, Ellerbrock TV, Chiasson MA, Bush TJ, Wright TCJ (1997). Human papillomavirus infection in women infected with the human immunodeficiency virus. N Engl J Med.

[B20] Conley LJ, Ellerbrock TV, Bush TJ, Chiasson MA, Sawo D, Wright TC (2002). HIV-1 infection and of risk of vulvovaginal and perianal condylomata accuminata and intraepithelial neoplasia: a prospective cohort study. Lancet.

[B21] Lillo FB, Ferrari D, Veglia F, Origoni M, Grasso MA, Lodini S, Mastrorilli E, Taccagni G, Lazzarin A, Uberti-Foppa C (2001). Human papillomavirus infection and associated cervical disease in human immunodeficiency virus-infected women: effect of highly active antiretroviral therapy. J Infect Dis.

[B22] Heard I, Schmitz V, Costagliola D, Orth G, Kazatchkine MD (1998). Early regression of cervical lesions in HIV-seropositive women receiving highly active antiretroviral therapy. AIDS.

[B23] Minkoff H, Ahdieh L, Massad LS, Anastos K, Watts DH, Melnick S, Muderspach L, Burk R, Palefsky J (2001). The effect of highly active antiretroviral therapy on cervical changes associated with oncogenic HPV among HIV-infected women. AIDS.

[B24] Richardson AT, Lyong JB (1981). The effect of condom use on squamous cell cervical intraepithelial neoplasia. Am J Obstet Gynecol.

[B25] Hogewoning CJ, Bleeker MC, van den Brule AJ, Voorhorst FJ, Snijders PJ, Berkhof J, Westenend PJ, Meijer CJ (2003). Condom use promotes regression of cervical intraepithelial neoplasia and clearance of human papillomavirus: a randomized clinical trial. Int J Cancer.

[B26] Branca M, Garbuglia AR, Benedetto A, Cappiello T, Leoncini L, Migliore G, Agarossi A, Syrjaren K (2003). Factors predicting the persistence of genital human papillomavirus infections and PAP smear abnormality in HIV-positive and HIV-negative women during prospective follow-up. Int J STD AIDS.

[B27] Santos C, Galdos R, Alverez M, Velarde C, Barriga O, Dyer R, Estrada H, Almonte M (1996). One session management of cervical intraepithelial neoplasia: a soloution for deveoping countries. A prospective, randomized trial of LEEP versus laser excisional conization. Gynecol Oncol.

[B28] Spitzer M, Chernys A, Seltzer V (1993). The use of large-loop excision of the transformation zone in an inner-city population. Obstet Gynecol.

[B29] Adam Y, Van Gelderen CJ, Newell K (2008). 'Look and Lletz'--a Chris Hani Baragwanath Hospital experience. S Afr Med J.

[B30] Xi LF, Kiviat NB, Wheeler CM, Kreimer A, Ho J, Koutsky LA (2007). Risk of cervical intraepithelial neoplasia grade 2 or 3 after loop electrosurgical excision procedure associated with human papillomavirus type 16 variants. J Infect Dis.

[B31] Hernadi Z, Szoke K, Sapy T, Krasznai ZT, Soos G, Veress G, Gergely L, Konya J (2005). Role of human papillomavirus (HPV) testing in the follow-up of patients after treatment for cervical precancerous lesions. Eur J Obstet Gynecol Reprod Biol.

[B32] Costa S, de Simone P, Venturolli S, Cricca M, Zerbini ML, Musiani M, Terzano P, Santini D, Cristiani P, Syrjänen S, Syrjänen K (2003). Factors predicting human Papillomavirus clearance in cervical intraepithelial neoplasia lesions treated by conization. Gynecol Oncol.

[B33] IARC (1995). Monographs on the evaluation of carcinogenic risks to humans: Human Papillomaviruses.

[B34] Reich O, Lahousen M, Pickel H, Tamussino K, Winter R (2002). Cervical intraepithelial neoplasia III: long-term follow-up after cold-knife conization with involved margins. Obstet Gynecol.

[B35] Costa S, De Nuzzo M, Infante FE, Bonavita B, Marinelli M, Rubino A, Rambelli V, Santini D, Cristiani P, Bucchi L (2002 ). Disease persistence in patients with cervical intraepithelial neoplasia undergoing electrosurgical conization. Gynecol Oncol.

[B36] Paraskevaidis E, Koliopoulos G, Malamou-Mitsi V, Zikopoulos K, Paschopoulos M, Pappa L, Agnantis NJ, Loli DE (2001 ). Large loop excision of the transformation zone for treating cervical intraepithelial neoplasia: a 12-year experience. Anticancer Res.

[B37] Nappi L, Carriero C, Bettocchi S, Herrero J, Vimercati A, Putignano G (2005). Cervical squamous intraepithelial lesions of low-grade in HIV-infected women: recurrence, persistence, and progression, in treated and untreated women. Eur J Obstet Gynecol Reprod Biol.

[B38] Maiman M, Fruchter RG, Serur E, Levine V, Arrastia CD, Sedlis A (1993). Recurrent cervical intraepithelial neoplasia in immunodeficiency virus-seropositive women. Obstet Gynecol.

[B39] Tate DR, Anderson RJ (2002). Recrudescence of cervical dysplasia among women who are infected with human immunodeficiency virus: a case-control analysis. Am J Obstet Gynecol.

